# The potential dangers of neck manipulation & risk for dissection and devastating stroke: An illustrative case & review of the literature

**DOI:** 10.15761/BRR.1000110

**Published:** 2018-03-25

**Authors:** Ryan C Turner, Brandon P Lucke-Wold, Sohyun Boo, Charles L Rosen, Cara L Sedney

**Affiliations:** 1 Department of Neurosurgery, West Virginia University School of Medicine, Morgantown, West Virginia; 2 Center for Neuroscience, West Virginia University School of Medicine, Morgantown, West Virginia; 3 West Virginia Clinical & Translational Science Institute, West Virginia University School of Medicine, Morgantown, West Virginia; 4 Department of Radiology, West Virginia University School of Medicine, Morgantown, West Virginia

**Keywords:** Chiropractic manipulation, Vertebral artery dissection, Brainstem infarct, Open communication, Medical clearance

## Abstract

Chiropractic cervical manipulation is a common practice utilized around the world. Most patients are never cleared medically for manipulation, which can be devastating for those few who are at increased risk for dissections. The high velocity thrust used in cervical manipulation can produce significant strain on carotid and vertebral vessels. Once a dissection has occurred, the risk of thrombus formation, ischemic stroke, paralysis, and even death is drastically increased. In this case report, we highlight a case of a 32-year-old woman who underwent chiropractic manipulation and had vertebral artery dissection with subsequent brainstem infarct. She quickly deteriorated and passed away shortly after arrival to the hospital. Although rare, one in 48 chiropractors have experienced such an event. We utilize this case to highlight the risk associated with cervical manipulation and urge open dialogue between chiropractors and physicians. Receiving medical clearance prior to cervical manipulation in potential at risk patients would drastically reduce morbidity and mortality.

## Introduction

Spinal manipulation, a hallmark practice of chiropractic providers and a practice occasionally utilized by physiotherapists, osteopaths, and physicians, is often viewed as a controversial and potentially dangerous procedure without good evidence supporting its use [1]. Specifically, epidemiologic associations have been observed between chiropractic maneuvers and cervical artery dissection, although it has been difficult to prove causal relationship [2–4]. The potential consequence of cervical artery dissection is stroke, which may have a range of consequences with regards to long-term morbidity and mortality [5–7].

Herein, we report a case of vertebral artery dissection and subsequent brainstem infarction immediately following chiropractic manipulation in a young female patient. This patient experienced rapid deterioration immediately following manipulation and proceeded to undergo cardiac arrest prior to presentation to the hospital and subsequent death. This case serves to highlight the potential dangers of chiropractic manipulation and provides a thorough review of the literature relating chiropractic manipulation and stroke. Furthermore, this case presentation demonstrates a likely causal relationship although prior imaging was not available to rule out a pre-existing dissection. The case therefore satisfies many of the criteria proposed by Hill [8] and used by Tuchin [9] to refute the role of chiropractic manipulation in dissection and stroke.

## Case Presentation

**Figure 1:**
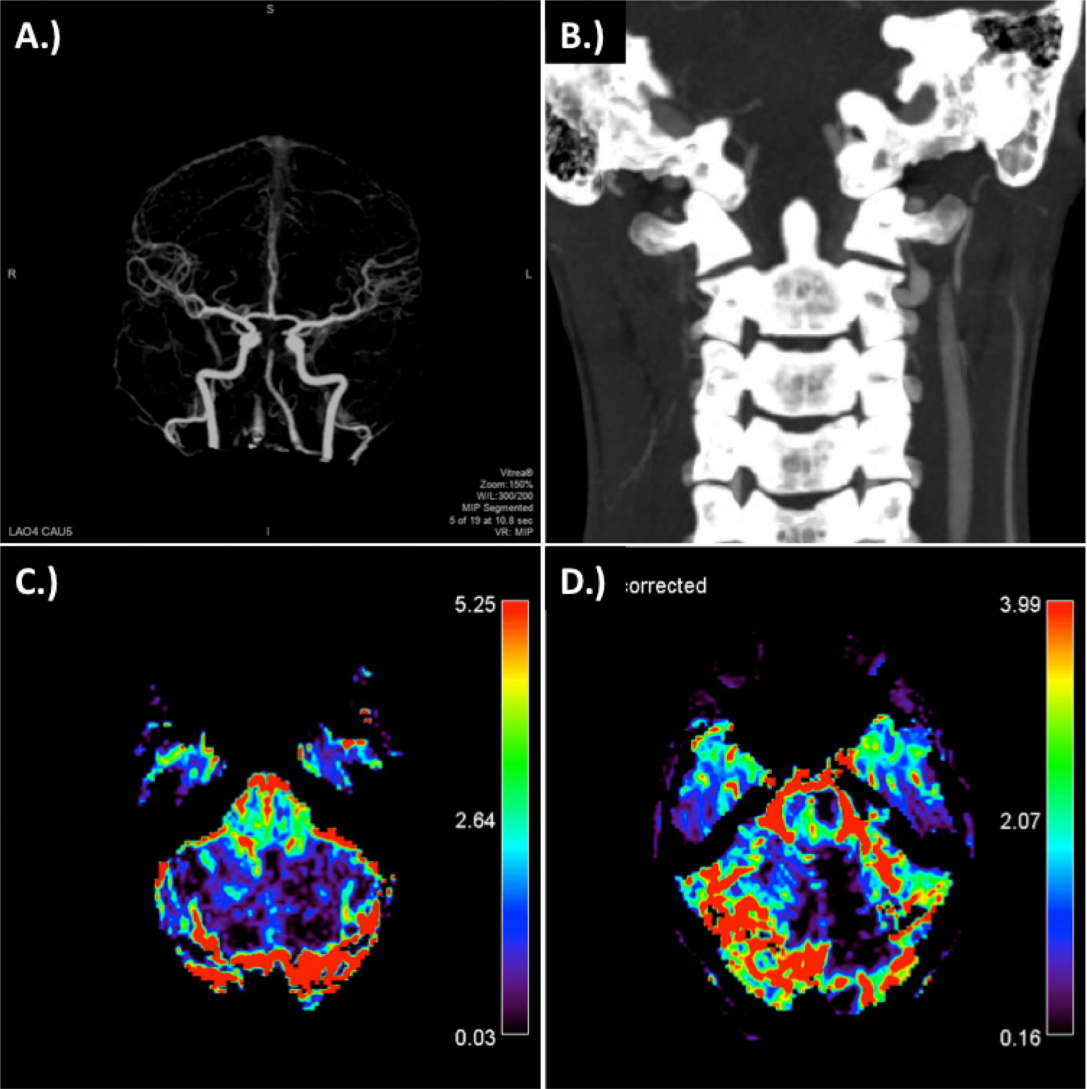
CT Stroke Protocol at time of presentation. A.) 4D CTA demonstrating top of the basilar occusion and poor flow in the right vertebral artery; B.) coronal CA demonstrating dissection of verterbral arteries at the C1–C2 level, particularly prominent on the right; C.) relative blood volume; and D.) corrected relative blood volume.

**Figure 2:**
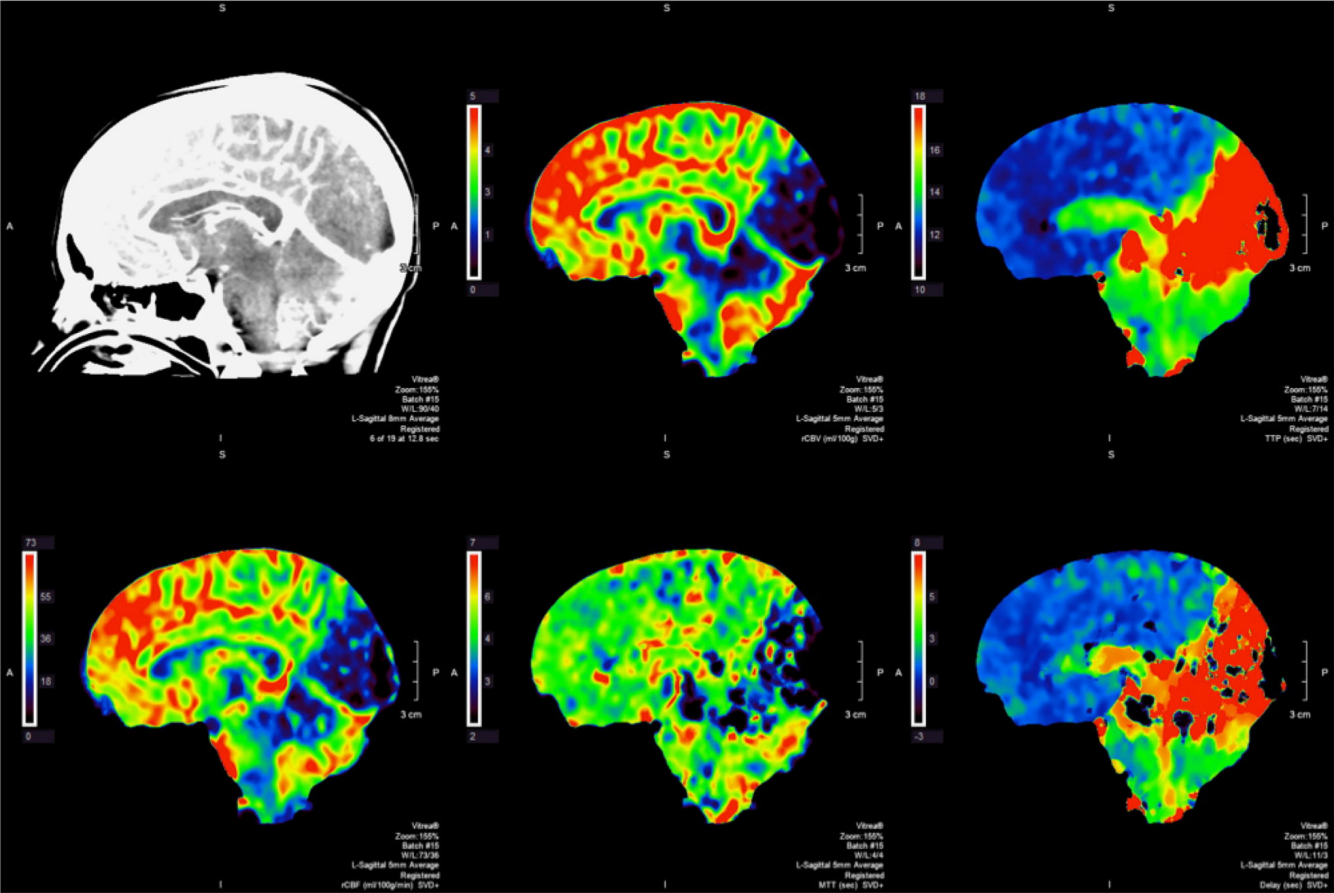
CT Stroke Protocol with perfusion imaging demonstrating primary involvement of occipital lobe, cerebellum, and brainstem. Perfusion imaging reflecting rCBV, TTP, rCBF, MTT, and delay (from top-left to bottom-right).

The patient, a 32-year-old Caucasian woman, presented to the West Virginia University Hospital Emergency Department via Emergency Medical Services. The patient had been at her usual baseline state of health with no significant past medical history prior to visiting the chiropractor for neck adjustment earlier that day for tension like soreness. The patient underwent neck manipulation after which she immediately complained of neck pain, diaphoresis, and proceeded to experience cardiac and respiratory arrest. Emergency Medical Services was called, and cardiopulmonary resuscitation was performed with one round of epinephrine administered. It was reported that the patient was pulseless and apneic for 3 minutes prior to EMS arrival. The patient was intubated on transport and her Glasgow Coma Scale score was 3T prior to arrival. Mean arterial blood pressure was 80 with palpable femoral pulses at arrival to the emergency department. Upon arrival in the emergency department, a CT stroke protocol was performed which demonstrated bilateral severe distal cervical vertebral artery dissections with acute thrombotic emboli seen in the left cervical vertebral artery (Figures 1 and 2). This was accompanied by complete occlusion of the basilar tip including the proximal posterior cervical arteries.

**Figure 3:**
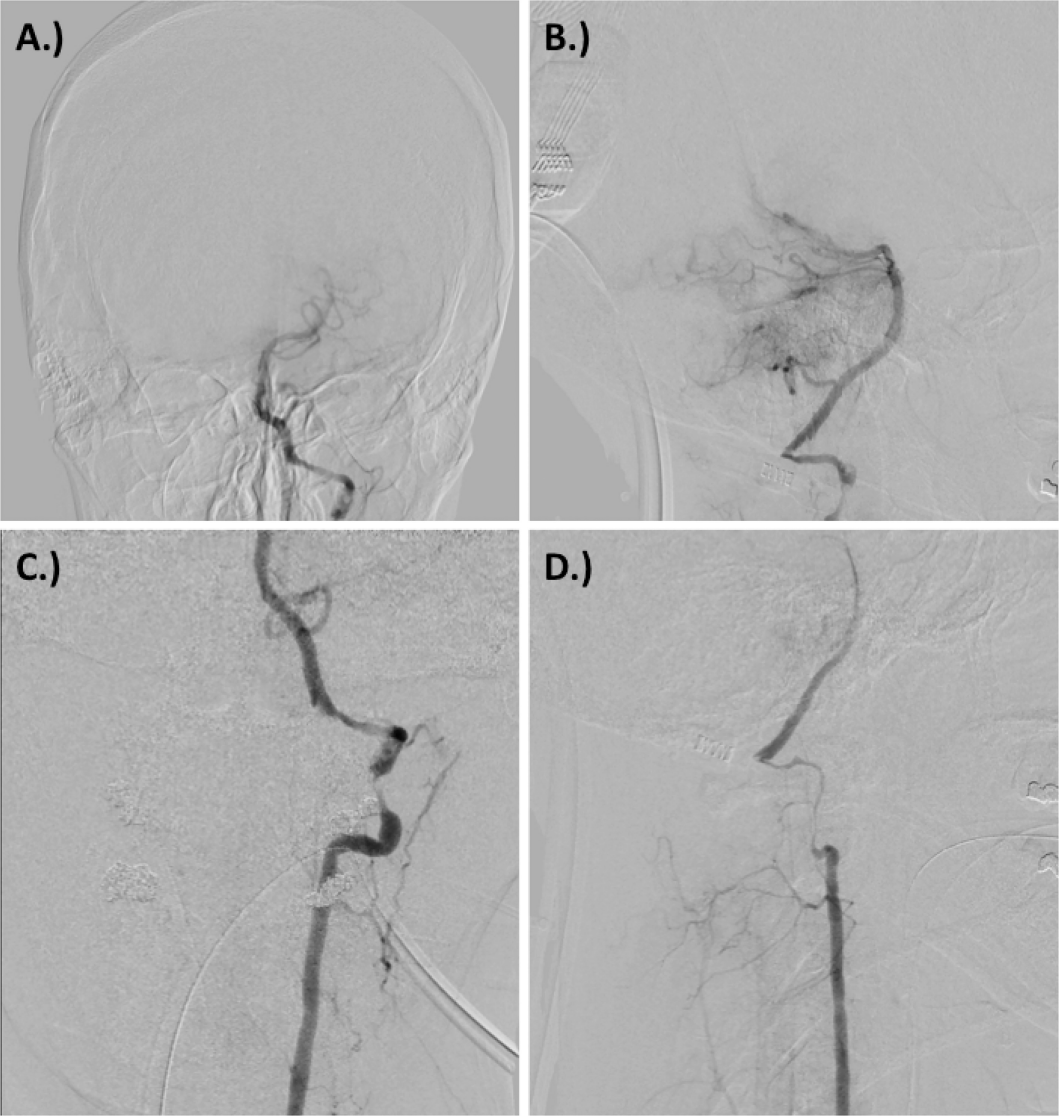
Digital subtraction angiography at time of presentation demonstrating A.) anterior-posterior view showing basilar and PICA occlusion, B.) lateral view showing basilar and PICA occlusion, C.) left vertebral artery dissection, and D.) right vertebral artery dissection.

The patient received an initial bolus of intravenous tissue plasminogen activator (IV rtPA) at this time and the decision was made to proceed with endovascular intervention given the recent onset of occlusion. The patient was brought to the neurovascular angiography suite and femoral access obtained. Angiography of the left vertebral artery demonstrated severe dissection involving the distal cervical vertebral artery segments at the C1-C2 level with presence of sub occlusive thrombi. There was an occlusive clot in the left Posterior Inferior Cerebellar Artery (PICA). Intracranial imaging demonstrated occlusion at the basilar apex with absent filling into the right Posterior Cerebral Artery (PCA). There was occlusion of the distal left PCA. Angiography of the right vertebral artery demonstrated severe dissection of the distal cervical vertebral artery at C1-C2 with the presence of trickle-like flow into the vertebrobasilar junction. No filling was observed in the right PICA territory (Figure 3). At this point, it was decided that the left vertebral artery offered the best access to the basilar trunk.

Subsequently, distal aspiration was begun with a Penumbra 5 Max ACE distal aspiration catheter which initially demonstrated slow flow through the suction tubing. The 5 Max ACE was withdrawn into the proximal basilar artery until flow was seen within the suction tubing. Repeat angiography at this time demonstrated recanalization of the basilar apex and proximal PCAs. TICI3 perfusion was seen in the right PCA. Occlusive clot remained in the left distal P2 segment. Given the large size of the PCA, timing of events, and patient’s age, the decision was made to attempt clot retrieval of this. At this time, the Trevo ProVue microcatheter was navigated into the left distal PCA distal to the clot. The Trevo 4 mm × 30 mm stent retriever was deployed for approximately 3 minutes. The suction canister was attached, and the stent retriever was pulled with distal aspiration. No significant recanalization was achieved with what amounted to TICI0 perfusion to the left PCA territory. No further attempts were made as it was believed that the left PCA territory had completed its infarction.

**Figure 4:**
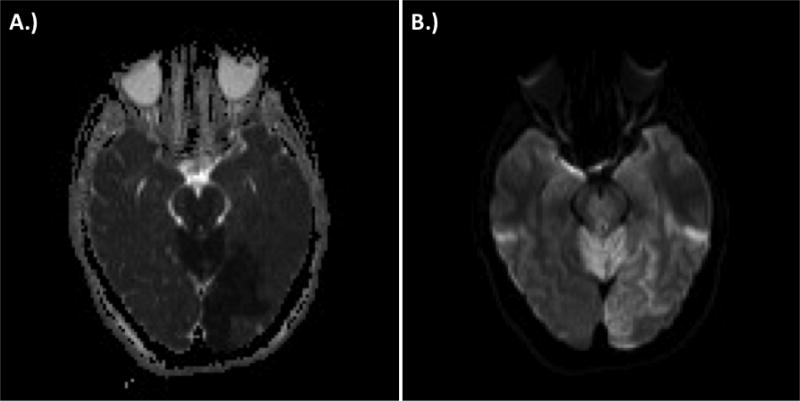
Immediate post-procedure magnetic resonance imaging demonstrating ischemic stroke within the cerebellum, brainstem, and left occipital cortex. A.) attenuated diffusion coefficient (ADC) sequence capturing left occipital stroke and B.) diffusion-weighted imaging capturing cerebellar, brainstem, and left occipital cortex stroke.

Following completion of endovascular therapy, the patient was taken for immediate MRI Brain with and without contrast for assessment of brainstem integrity and cerebrovascular status prior to transport to the intensive care unit. MRI demonstrated extensive areas of restricted diffusion accompanied by perfusion abnormalities consistent with acute infarction of the posterior circulation, specifically within the bilateral cerebellar hemispheres, right medulla, pons bilaterally, midbrain, thalami, and left occipital lobe (Figure 4). The following day, additional CT Brain imaging was acquired and demonstrated signs of elevation of intracranial pressure with hydrocephalus, worsening of cerebral edema diffusely, hemorrhagic transformation of the left occipital lobe, continued infarct evolution within the posterior circulation, and cerebellar tonsillar herniation.

## Discussion

Arterial dissection, particularly as related to the cervical arteries and risk of ischemic stroke, has become a topic of increasing interest in recent years. Many potential risk factors have been identified that include genetic disorders such as α_1_-antitrypsin (α1-AT) deficiency [10–12], connective tissue disease [13–15], MTHFR TT genotype [16], homocysteine concentration within the serum [16,17], history of migraine [18–20], variations in cardiac/cervical vessel anatomy [21–25], infection [10,19], oral contraceptive use [10,18,20], and trivial trauma [26–30]. A more rigorous analysis of these studies in the systematic review by Rubinstein and colleagues and additional completion of larger/more focused studies have failed to confirm that many of these variables are risk factors for dissection [31].

Cerebral and vertebral artery dissections can occur both at the chiropractor’s office and at home when practicing self-manipulation techniques [32]. Multi-artery dissection is a rare occurrence following chiropractic manipulation but can lead to extensive infarct and adverse outcomes [33]. A recent systematic review found that 901 cases of cerebral artery dissections have been reported in the literature in relation to chiropractic manipulation. In 707 of these cases, the patient went on to develop some type of stroke [34]. Notably, a study focused on malpractice data from the Canadian Chiropractic Protective Association (CCPA) showed a low incidence of neurological symptoms attributable to cervical manipulation and arterial dissection following chiropractic manipulation [35]. Specifically, data from the report suggests that a chiropractor will be aware of an arterial dissection occurring following cervical manipulation only once in 8.06 million office visits with only one in forty-eight chiropractors experiencing such an event in their entire careers [35]. Chiropractors are inexperienced in detecting the signs and symptoms of a dissection.

Diagnosing thrombosis early in these cases is critical for successful endovascular or surgical intervention [36]. For example, a recent case report showed that a patient with vertebral artery dissection and infarction following chiropractic manipulation was successfully treated with mannitol and ventriculostomy [37]. Unfortunately, despite early identification, cerebrovascular injury has resulted in at least 26 reported deaths following chiropractic spinal manipulation [38]. For our patient, clot formation was observed in the PICA, basilar apex, and distal P2 segment limiting endovascular and surgical options. Endovascular diagnosis and attempted treatment failed. The patient subsequently went on to develop hemorrhagic transformation, severe cerebral edema, and ultimately cerebellar tonsillar herniation. The key learning points for this case revolve around prevention.

Futch et al., 2015 urge chiropractic colleagues to have a high suspicion of vertebral artery dissection when patients come in for treatment with sudden neck pain and headache [39]. The use of magnetic resonance angiography in these cases may prevent the chiropractor from performing potentially dangerous manipulations in at risk individuals. Green and colleagues present modified manipulation techniques that don’t involve high velocity movements. These techniques might minimize the rotation of the neck and can potentially prevent vascular dissections [40]. In a cadaveric study, Piper and colleagues show that strain and peak angular displacement during spinal manipulation is highly variable between individuals. The peak strain often occurs at an intermediate point during the movement of the neck [41]. Therefore, even in experienced hands, a high velocity spinal manipulation may not be safe based on individual anatomical differences in patients

Based on the several reported cases of complications following spinal manipulation, we encourage increased communication between chiropractic and medical colleagues. A low threshold should exist for chiropractors to consult physicians in order to make sure the patient is cleared for manipulation. If the patient has been seen at a medical facility and MRI or CT images are suggestive of potential risks, this information needs to be communicated to the patient regarding heightened potential for adverse outcomes related to manipulation. Tarola and Phillips report a case where acute onset neck pain in a patient motivated the chiropractor to refer the patient to the emergency department before manipulation. MRA revealed vertebral artery dissection therefore preventing a potentially devastating outcome if manipulation had been performed [42]. In the future, mechanoreceptor data may be a viable option to investigate the safety of manipulation and thrust procedures on muscle spindle responses [43]. This neural based stimulation in conjunction with clinical judgment may be used to select patients at low risk for vascular injury.

## Conclusion

High velocity thrust manipulation of the cervical spine places the carotid and vertebral arteries at risk of dissection. Even with early diagnosis, thrombus occlusion and subsequent infarction can lead to serious adverse outcomes. We report a case of a young woman who had bilateral vertebral artery dissections following chiropractic manipulation. The patient had thrombus occlusion that was not amenable to endovascular treatment and developed hemorrhagic transformation, cerebral edema, and cerebellar herniation. Additionally, ongoing research is necessary to determine the safety of cervical manipulation and whether the techniques should be modified in order to prevent injury.
